# Effect of biochar on rice starch properties and starch-related gene expression and enzyme activities

**DOI:** 10.1038/s41598-020-73888-2

**Published:** 2020-10-09

**Authors:** Diankai Gong, Ximing Xu, Li’an Wu, Guijin Dai, Wenjing Zheng, Zhengjin Xu

**Affiliations:** 1 Shenyang Agricultural University, Shenyang, China; 2 Liaoning Rice Research Institute, Shenyang, China

**Keywords:** Physiology, Plant sciences

## Abstract

We determined the effects of biochar on starch properties and the activities of enzymes and expression levels of genes related to starch in two *Japonica* rice cultivars. The two rice varieties were subjected to five biochar treatments (0, control; and 5, 10, 20, and 40 t/hm^2^). In both rice varieties, the content of apparent amylose and resistant starch were lower in biochar treatments than in the control. The proportion of fa chains was higher and that of fb3 chain was lower in the biochar treatments than in the control. Starch viscosity and cooking taste quality were improved by the biochar treatments. In both rice varieties, the activity of granule-bound starch synthase was significantly decreased by biochar treatments, and the activities of soluble starch synthase, starch branching enzyme, and starch debranching enzyme were significantly increased. The transcript levels of genes encoding starch synthases and starch branching enzymes were significantly increased by biochar treatments. We conclude that biochar at a dose of 5–10 t/hm^2^ can regulate the activity of starch-related enzymes, and this affects the type, content, and fine structure of starch. Therefore, the addition of biochar to soil can improve the viscosity and taste quality of rice starch.

## Introduction

Biochar, generally refers to the carbon-rich solid product when biomass is thermally decomposed under anoxic conditions^1^. Most previous studies on biochar have focused on its interactions with soils, crops, and other factors in the environment. Research in many countries has shown that biochar can significantly improve soil structure and soil physical and chemical properties, modify the soil microbial community, and increase crop yields, and contribute to the prevention and control of air pollution, reductions in carbon dioxide emissions, and the sustainable development of agriculture^2–4^. Many studies have explored the effects of adding biochar to rice fields on yield, soil and greenhouse gases, but few have focused on its effects on rice quality and the related mechanisms.

Starch is the most important component of rice grains, and and the main factor affecting rice cooking and eating quality^5,6^. Starch accumulates in rice grains via a series of complex enzymatic processes. Rice starch is formed in the chloroplast as a product of photosynthesis, and then is decomposed into dihydroxy-phosphate, which is transported from the chloroplast to the matrix where it is transformed into sucrose via the activities of sucrose phosphate synthase (SPS), sucrose synthase (SuSy), and acid invertase (AI). The sucrose is then transported to reservoir organs to provide materials for starch synthesis. The sucrose entering the grain cell sap forms adenosine diphosphate glucose (ADPG) through glycolysis, then amylose is formed*via* the activity of granule-bound starch synthase (GBSS). Then, amylopectin is formed via the combined activities of soluble starch synthase (SSS), starch branching enzyme (SBE) and starch debranching enzyme (SDBE). Starch synthases can be divided into four types: SS I–IV. The SS I-type mainly combines A and B1 chains with very short external segments with a degree of polymerization (DP) of 6–7 to form DP 8–12 segments^7^. The SS IIa-type is responsible for the formation of medium-length branch chains (i.e., the addition of DP 6–11 to DP 13–28 chains)^8^, while SS IIIa participates in the formation of long chains (DP ≥ 30), particularly B2 and B3 chains^9^. Previous studies have shown that the expression levels of genes encoding products related to starch synthesis and metabolism are regulated by environmental factors and cultivation conditions, and that changes in the composition of amylose and amylopectin affect the cooking and taste qualities of rice^10,11^. In this experiment, two Japonica rice varieties, SN265 and Japanese Akihikari were used to study the effects of adding biochar to soil at different concentrations on the activities of key enzymes involved in sucrose metabolism in seeds and on the relationship between starch quality and the transcript levels of starch-related genes (*SS I*, *SS IIa*, *SS IIIa*, *SBE I*, *SBE IIb*, *ISA I* and *GBSS I*). The overall aim of this research was to explore the molecular mechanism by which biochar affects the properties of rice starch.

## Materials and methods

### Plant materials and experimental design

We conducted a pot experiment using the rice varieties ShenNong265 (hereafter, SN265) and Japanese Akihikari (hereafter, Akihikari). This experiment was carried out at Shenyang, Liaoning Province, China, from 2018 to 2019. Rice husk biochar was added to soil at five rates: 0 t/hm^2^ (B1, control), 5 t/hm^2^ (B2), 10 t/hm^2^ (B3), 20 t/hm^2^ (B4) and 40 t/hm^2^ (B5). The bulk density of the soil in this area is 1.34 g/cm^3^, and the soil quality was calculated for soil in the top 15-cm layer. When 10 t/hm^2^ of biochar was applied, the mass ratio in the soil was 5‰. Based on this calculation, the amounts of soil and biochar in the control and treatments were as follows: Control (B1): 15 kg soil per pot ; B2: 75 g biochar + 14.93 kg soil per pot; B3: 150 g biochar + 14.85 kg soil per pot; B4: 225 g biochar + 14.78 kg soil per pot; B5: 300 g biochar + 14.70 kg soil per pot. The soil and biochar were mixed before adding to each pot (pot diameter × height = 35 cm × 30 cm). The rice seeds were sown on April 15, with two seeds in per hole for plate seedling cultivation. The seedlings were raised in a greenhouse, and were transplanted at the 3.5-leaf stage. At this stage, seedlings with the same height were planted with equal spacing in the pots, with 1 seedling per hole, 3 holes per pot, and 16 pots per treatment. In total, 15 kg of nitrogen fertilizer (calculated based on pure nitrogen) was applied in three applications (basal: tiller: ear, 5:4:1). Irrigation was managed as per the normal practices in the area. The biochar was made from rice husk (particle size 1.5–2.0 mm), and was produced and provided by the Liaoning Jinhefu Agricultural Development Co., Ltd. The physical and chemical properties of potting soil and biochar are shown in Table 1. Samples with uniform growth length were taken from every treatment at 10 days after heading. All samples were stored at − 80 °C until use. At harvest, all samples from one pot were mixed as one replicate before threshing. The brown rice grains were dried naturally for 3 months and then processed. After sieving through a 1.7 mm classifier, the polished rice was ground with a full-automatic rice mill.

**Table 1:** Initial soil and rice husk biochar properties.

	pH	Total N%	Total P%	Total K%	Available N (mg/kg)	Available phosphorus (mg/kg)	Available potassium (mg/kg)	Available silicon (mg/kg)	Exchangeable calcium (mg/g)	Exchangeable magnesium (mg/g)	Organic matter (g/kg)
Soil	6.79	0.144	0.064	1.64	124.72	63.41	160.96	180.5	5.30	0.67	19.5
Biochar	9.32	0.953	0.110	1.69	49.68	52.30	106.83	237.3	0.55	0.20	–

### Measurement of physical and chemical properties of rice

The rice appearance quality was determined using Wanshen SCE rice appearance detection analysis software. The eating quality was measured using a Satake rice taste analyzer (SATAKE Corp., Hiroshima, Japan)., The amylose content in rice was determined by the sulfuric acid anthracene copper method^12^. The amylopectin side chain branch content was determined using the method of Nakamura et al.^17^. A Rapid Visco Analyzer (RVA; NEWPORT SCIENTIFIC Pty. Ltd, Warriewood, Australia) was used to test the viscosity of rice flour starch in the samples in accordance with the standards^13^ specified by the regulated by American Association of Cereal Chemists, Analyses were conducted using TCW (Thermal Cycle Windows) software.

### Activities of starch-related enzymes and transcript levels of their encoding genes

The activities of GBSS, SSS, SBE and DBE in rice grains were measured using the methods described by Jin Zhengxun et al.^14^. To measure gene transcript levels, total RNA was extracted by the Trizol method from the rice grains stored at − 80 °C. Then, the total RNA was treated with DNase *I* to eliminate DNA and cDNA was synthesized using a reverse transcription kit (Nova, purchased from Jiangsu Yugong Biolabs Inc). According to the gene RNA sequence published at the NCBI (https://www.ncbi.nlm.nih.gov), qRT-PC primers (Table 2) were designed with software Premier 5.0, and BLAST comparisons were made at the NCBI website to ensure the specificity of the primers. With cDNA as the template and *Actin1* as the internal reference gene, qRT-PCR analyses were conducted using a Taq SYBR Green qPCR kit. Each sample was analyzed three times. The relativetranscript levels of the target genes were calculated using the ΔCt method^15^.

**Table 2:** Target gene and sequence of qRT-PCR primers.

Gene name	Forward primer (5′–3′)	Reverse primer (5′–3′)
*Actinl*	TCTCAACCCCAAGGCCAATC	ATGAGTAACCACGCTCCGTC
*SSI*	GGGCCTTCATGGATCAACC	CCGCTTCAAGCATCCTCATC
*SSIIa*	GGCCAAGTACCAATGGTGAA	GCATGATGCATCTGAAACAAAGC
*SSIIIa*	GCCTGCCCTGGACTACATTG	GCAAACATATGTACACGGTTCTGG
*GBSSI*	GATGAGATACGGAACGCCCT	GCCCATGTGGAAACCAGTCT
*ISA*	GGGTCAATTTCGCCGTCTAC	CATTCCCCGTCCGATTGAAC
*SBEI*	TGGCCATGGAAGAGTTGGC	CAGAAGCAACTGCTCCACC
*SBEIIb*	ATGCTAGAGTTTGACCGC	AGTGTGATGGATCCTGCC
*AGPL2*	TAGATAGGCCTTGGAATCGCACC	TAGAGTTCCCATTCCAAAACAAACC
*AGPS2*	TCTTTTGTTGCCCATTCATCTGG	TGATTCCAAGCACACTCTCATCGAC

### Data analysis

The data were processed and analyzed using GraphPad Prism 8 and SPSS 18 statistical software. ANOVA and Duncan’s tests were used to detect significant differences among treatments.

## Results and analysis

### Effect of biochar on rice appearance and processing quality

As shown in Table 3 the biochar treatments directly affected the rate of chalky rice and the chalkiness degree of rice in both SN265 and Akihikari. For both varieties, the rate of chalky rice and the chalkiness degree were significantly lower in the B2 and B3 treatments than in the control. There was no significant difference in the length–width ratio of the seeds among treatments. For both rice varieties, the rate of the coarse was higher in B2 and especially B3, but the differences among the other treatments and between B2/B3 and the control were small. For SN265, the rates of polished rice and head rice were significantly higher in the B2 and B3 treatments than in the control, but not significantly different between the other treatments and the control. For Akihikari, the rates of polished rice and head rice rate were also higher in the B2 and B3 treatments than in the control and the other treatments, but this difference was not statistically significant.

**Table 3:** Effect of biochar on rice appearance and processing quality.

Variety	Treatment	chalky rice rate	chalkiness degree	Length–width ratio	Coarse rice rate	Polished rice rate	Head rice rate
SN265	B1	14.93a	3.46b	1.78a	80.59bc	71.83b	67.72c
	B2	11.62b	3.01b	1.77a	81.08ab	72.97a	69.9ab
	B3	10.71b	2.71b	1.79a	81.28a	72.99a	70.39a
	B4	12.54ab	3.34b	1.80a	80.51c	72.46ab	68.7bc
	B5	14.90a	4.71a	1.79a	80.30c	71.93b	68.15c
Akihikari	B1	27.73ab	10.00a	1.75a	82.02b	72.5ab	62.51ab
	B2	17.48c	5.37c	1.70a	82.89ab	73.76a	63.84a
	B3	18.78c	6.03c	1.73a	83.45a	74.45a	64.20a
	B4	25.67b	8.18b	1.75a	83.08ab	74.01a	61.87ab
	B5	28.60a	9.25a	1.73a	82.29ab	71.64b	60.21b

Different letters indicate significant difference at 5% level.

### Effect of biochar on rice starch properties and eating quality

#### Effect of biochar on rice starch composition and viscosity

As shown in Table 4 that the apparent amylose and resistant starch content of the two rice varieties decreased to a certain extent in response to biochar treatments. The apparent amylose and resistant starch contents of Akihikari were higher in the B4 treatment than in the control, but tended to be lower in other treatments, especially those in B3, than in the control. In both rice varieties, the proportion of Fa chains was significantly increased in the biochar treatments compared with the control, with only small differences among the biochar treatments. The proportion of Fb3 chains was lower in the biochar treatments than in the control, with only small differences among the biochar treatments. Therefore, in most cases, the Fa/Fb3 ratio was significantly higherin the biochar treatments than in the control. The Fa/Fb3 ratio was significantly higher in the B3 treatment than in the other treatments in Akihikari, but not in SN265.

**Table 4:** Effect of biochar on rice starch composition.

Variety	Treatment	Apparent amylose%	Resistant starch%	Fa%	Fb3%	Fa/Fb3
SN265	B1	17.24a	3.01a	22.87b	15.69a	1.47c
	B2	16.67b	2.77b	23.19ab	15.27b	1.52bc
	B3	16.05c	2.61c	23.55a	14.81c	1.59a
	B4	16.63b	2.65bc	23.40a	15.11bc	1.55ab
	B5	16.88ab	2.65bc	23.36a	15.16b	1.55ab
Akihikari	B1	16.4a	2.67a	23.24c	15.08a	1.54c
	B2	15.50bc	2.44b	23.68ab	14.58b	1.63b
	B3	14.99c	2.19c	23.84a	14.13c	1.69a
	B4	15.89ab	2.53b	23.47bc	14.78b	1.59b
	B5	15.57b	2.51b	23.56b	14.64b	1.61b

Different letters indicate significant difference at 5% level.

Table 5 shows the viscosity, hot pulp viscosity, disintegration value and cold glue viscosity of starch from the two rice varieties in the various treatments. The values of these parameters tended to be higher in the biochar treatments, especially B2 and B3, than in the control. The differences in these values among the other treatments and between the other treatments and the control were small. There were no significant differences in peak time and gelatinization temperature among the different biochar treatments among the treatments and between the treatments and the control.

**Table 5:** Effect of biochar on rice starch viscosity.

Variety	Treatment	Highest viscosity	Hot pulp viscosity	Disintegration value	Cold glue viscosity	Peak time	Gelatinization temperature
SN265	B1	2789.83c	2018.00b	771.83b	2991.83d	6.41a	71.40a
	B2	2903.00b	2063.5ab	839.50a	3061.50c	6.44a	71.02a
	B3	2981.67a	2121.67a	860.00a	3138.33a	6.41a	71.00a
	B4	2904.00b	2103.67a	800.33b	3076.33b	6.45a	70.99a
	B5	2824.83c	2032.67b	792.17b	3024.67 cd	6.41a	71.41a
Akihikari	B1	2792.67c	2129.17c	663.50c	3157.50b	6.58a	72.78a
	B2	2919.00a	2189.67ab	741.00a	3203.83a	6.52a	71.88b
	B3	2936.33a	2221.83a	714.50b	3209.83a	6.57a	72.18b
	B4	2868.67b	2164.83bc	703.83b	3185.83ab	6.57a	71.93b
	B5	2829.33c	2130.67c	698.67b	3154.00b	6.50a	72.75a

Different letters indicate significant difference at 5% level.

#### Effect of biochar on taste quality of rice

As shown in Table 6, for SN265, the treatments could be ranked, from highest to lowest eating value, appearance, viscosity and balance degree, as follows: B3 > B2 > B1, B4 and B5. The hardness was significantly lower B3 than in B2, and the hardness of B3 and B2 was significantly lower than that of the control and the other treatments. The hardness of B4 and B5 was similar to that of the control. For Akihikari, the treatments could be ranked, from highest to lowest taste value, appearance and balance degree, as follows: B3, B2 > B4, B5 > B1. For the viscosity, the treatments were ranked as follows: B3 > B2 > B5, B4, B1. The hardness showed no significant difference among the biochar treatments, but was significantly lower in the biochar treatments than in the control.

**Table 6:** Effect of biochar on taste quality of rice.

Variety	Treatment	Eating value	Appearance	Hardness	Viscosity	Balance
SN265	B1	42.78c	2.17b	8.48ab	2.65c	2.25c
	B2	45.12b	2.60a	8.27c	2.95b	2.63b
	B3	48.07a	2.82a	8.07d	3.51a	3.13a
	B4	42.33c	2.12b	8.55a	2.68c	2.20c
	B5	42.62c	2.22b	8.43b	2.60c	2.27c
Akihikari	B1	43.55d	2.43c	8.32a	2.57c	2.37d
	B2	51.18ab	3.60a	8.03b	4.12b	3.58b
	B3	53.28a	3.88a	8.00b	4.67a	3.92a
	B4	45.75c	2.90b	7.98b	2.65c	2.77c
	B5	45.50c	2.80b	8.03b	2.67c	2.73c

Different letters indicate significant difference at 5% level.

### Effect of biochar on activity of rice starch related enzymes and transcript levels of their encoding genes

#### Effects of biochar on starch-related enzyme activity

**Figure 1:**
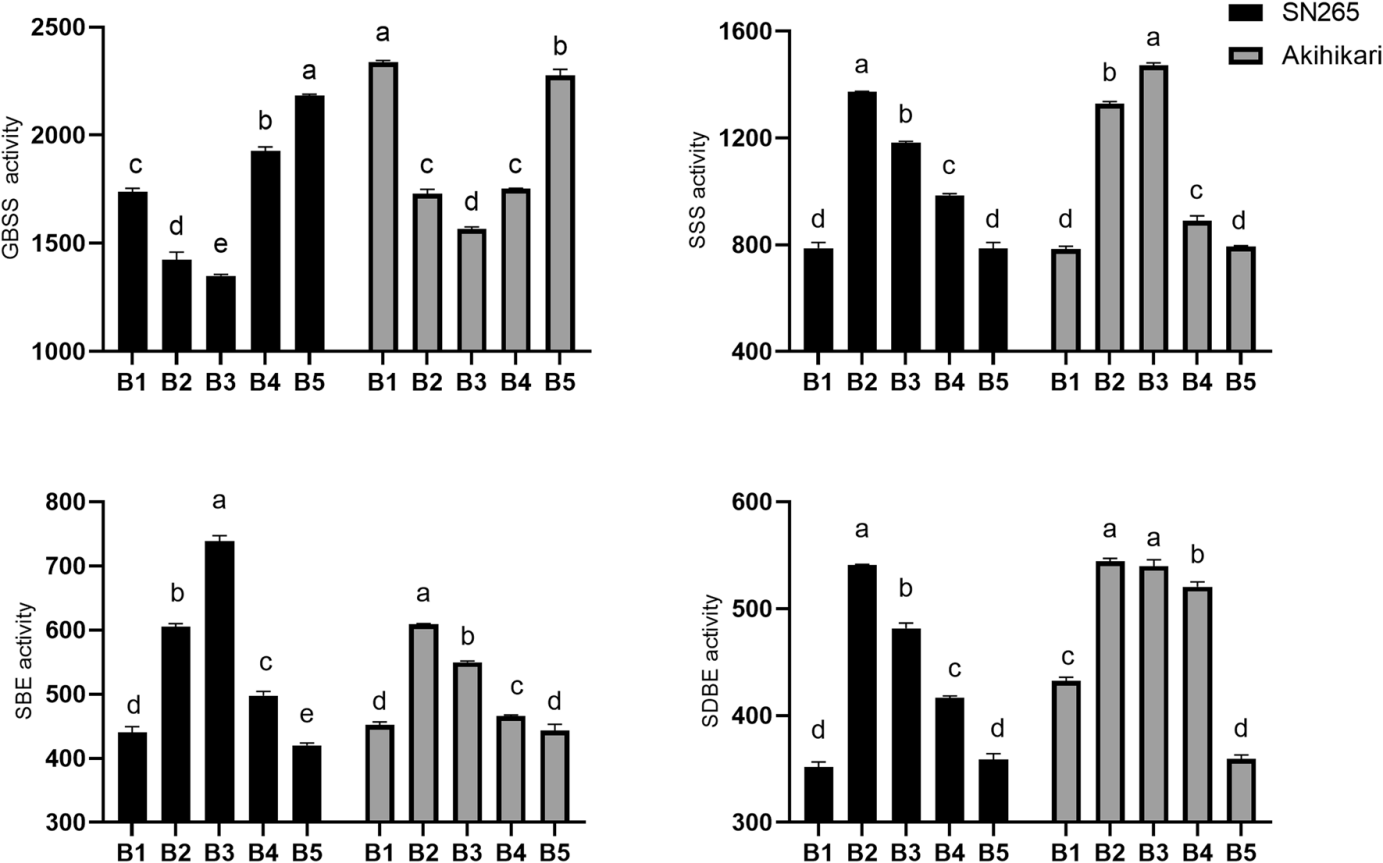
Effects of biochar on activities of starch-related enzymes in grains of two *Japonica* rice varieties, SN265 and Akihikari. Abbreviations: GBSS, granule-bound starch synthase; SSS, soluble starch synthase; SBE, starch branching enzyme; SDBE, starch debranching enzyme. Biochar treatments: 0 t/hm^2^ (B1, control), 5 t/hm^2^ (B2), 10 t/hm^2^ (B3), 20 t/hm^2^ (B4) and 40 t/hm^2^ (B5). Different letters indicate significant difference at 5% level.

The treatments could be ranked, from highest GBSS activity to lowest, as follows: B5 > B4 > B1 > B2 > B3 (SN265) and B1 > B5 > B2, B4 > B3 (Akihikari). The common feature was that GBSS activity was significantly lower in B3 and B2 than in the control, and lower in B3 than in B2 (Fig. 1). The activity of SSS was significantly higher in B2, B3 and B4 than in the control and B5, and significantly higher in B2 and B3 than in B4. The trends in SBE and SDBE activities in both rice varieties were similar to that of SSS, but in Akihikari, the activity of SDBE was slightly higher in B2 and B3 than in B4.

#### Effect of biochar on transcript levels of starch-related genes in rice grains

**Figure 2:**
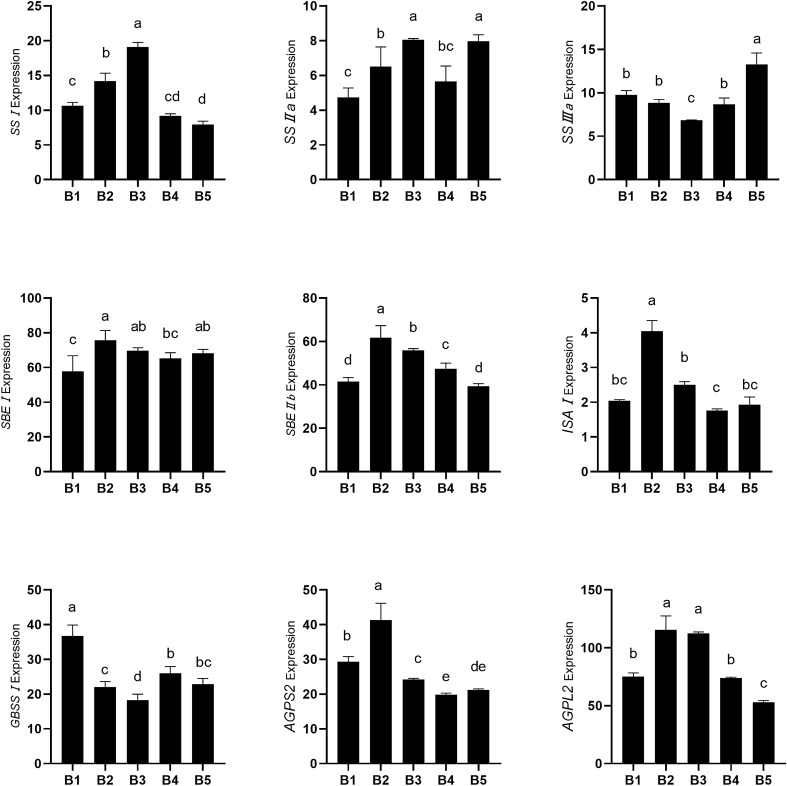
Effect of biochar on transcript levels of starch-related genes in rice grains. Gene transcript levels were calculated relative to that of *Actin1*. Biochar treatments: 0 t/hm^2^ (B1, control), 5 t/hm^2^ (B2), 10 t/hm^2^ (B3), 20 t/hm^2^ (B4) and 40 t/hm^2^ (B5). Different letters indicate significant difference at 5% level.

As shown in Fig. 2, the transcript levels of *SS I* were higher in B3 than in B2, and higher in both of those treatments than in the other biochar treatments. The differences in *SS I* transcript levels between B1 and B4 and between B4 and B5 were not significant. The trends in *SS II* transcript levels were similar to those of *SS I*, but there was no significant difference in *SS II* transcript levels between B5 and B3. The treatments could be ranked, from highest *SS III* transcript level to lowest, as follows: B5 > B1, B2, B4 > B3. For *SBE I,* its transcript levels were higher in the biochar treatments than in the control, and those differences were significant for all treatments except for B4. The treatments could be ranked, from highest transcript level of *SBE IIb* to lowest, as follows: B2 > B3 > B4 > B1, B5. The transcript levels of *ISA* were significantly higher in B2 than in the other treatments, but the differences among the other treatments were not statistically significant. The transcript levels of *GBSS I* were significantly lower in the biochar treatments, especially B3, than in the control. The rank order of the treatments in terms of *AGPS2* transcript levels was B2 > B1 > B3 > B4, B5, and in terms of *AGPL2* transcript levels it was B2, B3 > B1, B4 > B5.

## Conclusion and discussion

The key to the eating quality of rice is viscoelasticity and other texture characteristics of rice. The starch content in the rice endosperm plays an important role in rice cooking and eating quality^16,17^. Endosperm starch consists of two kinds of α-glucose chains with different structures, namely amylose and highly branched amylopectin^14^. Rice with low amylose content is sticky and soft, while rice with high amylose content is relatively loose and hard after cooking^18–21^. The proportions of amylose and amylopectin and the fine structure of amylopectin affect the physical and chemical properties and RVA characteristics of starch, thereby affecting the processing quality and eating quality of rice grains. Amylopectin can be classified according to branch length into short-chain Fa, medium-chain Fb1 and Fb2, and long-chain Fb3. Many studies have shown that amylose content is the most important factor in rice quality^18^, and it has been used to predict the cooking and eating quality of rice for many years^16^. Most high-quality rice varieties have a low or medium amylose content. According to previous studies, the lower the AAC%, the greater the viscosity and the lower the hardness of rice, which result in better the palatability is. Allahholipour^22^ found that rice varieties with good palatability have a low AAC%, a high disintegration value, and low a cooling value. Li^18^ showed that a high proportion of amylose and long amylopectin branches lead to greater elasticity and lower viscosity; a high proportion of long amylopectin Fb3 increases hardness, and an increase in Fa content and Fa/Fb3 lead to higher viscosity. In our study, we found that biochar can reduce the apparent amylose content of SN265 and Akihikar, and increase the Fa content and Fa/Fb3 ratio in rice starch, especially the 10 t/hm^2^ biochar treatment. This ultimately improved the viscosity and taste quality of both rice varieties.

Starch accumulates via a complex series of enzymatic processes. Rice starch is formed in the chloroplast from the products of photosynthesis, and amylose is formed via the activity of GBSS, the key enzyme for amylose synthesis in the endosperm^23–25^. It is encoded by *GBSS I* whose transcript levels directly affect amylose content. Thus, inhibiting *GBSS I* expression can reduce the amylose content in seeds^26^. Amylopectin is produced under the combined actions of SSS, SBE, and DBE. SSS extends glucose chain linked by α-1,4 glucoside, and then branches are added by the activity of SBE. Finally, DBE removes the unsuitable branches to synthesize amylopectin. In the rice endosperm, SBE *I* and SBE *II* are responsible for the synthesis of 70% and 30% of amylopectin, respectively^27–29^. SBE I and SBE IIIb play different roles in the formation of the two types of branches: SBE *I* tends to transfer longer branched chains, and provides the substrate for SS *III*a to generate medium-sized and long chains, In contrast, SBE *II*b tends to utilize short chains (DP 6–7) , which are subsequently extended by SS *I*^17,29^. The starch de-branching enzyme ISA uses amylopectin as its specific substrate, which is directly involved in the synthesis of amylopectin. ISA *I* is one of the most important genes determining starch structures in rice grains^10,30,31^. In this study, we found that biochar treatments affected the fine structure and content of starch by regulating the expression of genes encoding products related to starch synthesis and modification. This ultimately affected the viscosity and eating quality of rice. With increasing amounts of biochar added to soil, the transcript levels of *GBSS I* first decreased and then increased, and the activity of GBSS also first decreased and then increased. Consequently, the amylose content was significantly lower in B3 than in the other treatments. The transcript levels of *SS IIa* and *SS IIIa* affected the activity of amylase. The transcript level of *SS IIa* was significantly higher in B3 than in the control.The transcript level of *SS IIIa* was lowest in B3, resulting in the highest proportion of Fa chains and the lowest proportion of Fb3 chains, which affected the Fa/Fb3 ratio. The highest relative transcript levels of *SBE I*, *SBE IIb* and *ISA I* were in B2, much higher than in the control.

Our results show that biochar affected the expression of genes related to starch synthesis, which led to changes in the activities of enzymes involved in the synthesis of amylose and amylose, thereby changing the quality of rice. We propose two possible mechanisms for this effect: (1) There are many water-soluble active molecules on the surface of biochar. These include organic compounds such as carboxylic acid, acetoxy acid, benzoic acid, glycol, triol and phenolic substances^32^. Active molecules enter the plant body directly through the root system. After the active molecule enters the plant, it binds to the key protein/gene involved in starch synthesis, and then activates/inhibits a new metabolic pathway that ultimately affects grain quality. Previous studies have found that water-soluble molecules from biochar can alter the transcript levels of genes to improve drought and salt resistance in rice^33,34^. To explore this idea, we intend to use modern molecular biology techniques to screen and detect target genes or new metabolic pathways in further studies. (2) Biological carbon (especially rice husk carbon) contains abundant silicon that can be used directly and indirectly by plants. Many studies have shown that silicon can improve photosynthesis and promote carbohydrate synthesis and transport^35^. Silicon can also improve grain appearance quality and total starch content, and reduce amylose content and chalkiness to improve grain quality^36–39^. Based on the results of this study, we propose that an appropriate amount of biochar can increase the expression of genes and the activity of enzymes related to starch synthesis, which affects the types and content of starch and ultimately affects rice quality. The specific mechanism of its upstream function is unknown, and will be explored in further research.

Under the conditions of this study, the addition of 5–10t/hm^2^ biochar to improved the taste quality of rice, and also improved its appearance and processing quality. To reduce costs while still achieving tangible benefits, the rate of 5t/hm^2^ biochar is recommended. According to the estimated 30–40% yield of biochar from straw, 5 t/hm^2^ biochar is approximately equivalent to the carbonization of the full amount of straw, which is feasible for production. Given the stable long-term effectiveness of biochar, measures such as carbonizing straw every other year, or reducing the amount of biochar after continuous application, are proposed.

## Conclusion

A certain amount of biochar can regulate the activities of enzymes related to starch synthesis and metabolism by affecting the transcript levels of their encoding genes. These changes affect the content and fine structure of starch in rice grains, and ultimately improve the viscosity characteristics and taste quality of starch, as well as the appearance and processing quality of rice grains. Our results indicate that an appropriate rate of biochar addition is 5–10 t/hm^2^. Further studies are required to clarify the detailed physiological, biochemical, and molecular biological mechanisms underlying the effect of biochar to improve the taste, appearance and processing of rice.
